# High Density Linkage Map Construction and Mapping of Yield Trait QTLs in Maize (*Zea mays*) Using the Genotyping-by-Sequencing (GBS) Technology

**DOI:** 10.3389/fpls.2017.00706

**Published:** 2017-05-08

**Authors:** Chengfu Su, Wei Wang, Shunliang Gong, Jinghui Zuo, Shujiang Li, Shizhong Xu

**Affiliations:** ^1^Department of Life Sciences, Liupanshui Normal UniversityLiupanshui, China; ^2^Department of Botany and Plant Sciences, University of California, RiversideRiverside, CA, USA; ^3^Department of Economic Crop, Agricultural Science Institute of Coastal Region of JiangsuYancheng, China; ^4^Institute of Grain and Oil, Liupanshui Academy of Agricultural SciencesLiupanshui, China

**Keywords:** bin map, genotyping by sequencing, maize, quantitative trait loci, yield

## Abstract

Increasing grain yield is the ultimate goal for maize breeding. High resolution quantitative trait loci (QTL) mapping can help us understand the molecular basis of phenotypic variation of yield and thus facilitate marker assisted breeding. The aim of this study is to use genotyping-by-sequencing (GBS) for large-scale SNP discovery and simultaneous genotyping of all F_2_ individuals from a cross between two varieties of maize that are in clear contrast in yield and related traits. A set of 199 F_2_ progeny derived from the cross of varieties SG-5 and SG-7 were generated and genotyped by GBS. A total of 1,046,524,604 reads with an average of 5,258,918 reads per F_2_ individual were generated. This number of reads represents an approximately 0.36-fold coverage of the maize reference genome Zea_mays.AGPv3.29 for each F_2_ individual. A total of 68,882 raw SNPs were discovered in the F_2_ population, which, after stringent filtering, led to a total of 29,927 high quality SNPs. Comparative analysis using these physically mapped marker loci revealed a higher degree of synteny with the reference genome. The SNP genotype data were utilized to construct an intra-specific genetic linkage map of maize consisting of 3,305 bins on 10 linkage groups spanning 2,236.66 cM at an average distance of 0.68 cM between consecutive markers. From this map, we identified 28 QTLs associated with yield traits (100-kernel weight, ear length, ear diameter, cob diameter, kernel row number, corn grains per row, ear weight, and grain weight per plant) using the composite interval mapping (CIM) method and 29 QTLs using the least absolute shrinkage selection operator (LASSO) method. QTLs identified by the CIM method account for 6.4% to 19.7% of the phenotypic variation. Small intervals of three QTLs (*qCGR-1, qKW-2*, and *qGWP-4*) contain several genes, including one gene (GRMZM2G139872) encoding the F-box protein, three genes (GRMZM2G180811, GRMZM5G828139, and GRMZM5G873194) encoding the WD40-repeat protein, and one gene (GRMZM2G019183) encoding the UDP-Glycosyltransferase. The work will not only help to understand the mechanisms that control yield traits of maize, but also provide a basis for marker-assisted selection and map-based cloning in further studies.

## Introduction

Maize (*Zea mays*) is one of the most important cereal and forage crops of the world. As a result, high grain yield is a constant topic and pursuing direction of maize breeders. Most yield related traits are quantitative in nature and are often controlled by multiple genes. Grain yield of maize is a complicated agronomic trait that is mainly determined by 100-kernel weight (KW), ear length (EAL), ear diameter (EAD), cob diameter (CD), kernel row number (KRN), corn grains per row (CGR), ear weight (EW), and grain yield per plant (GWP). Quantitative trait loci (QTL) mapping has been successfully applied to maize and with this technology people have identified many loci relevant to yield and yield component traits (Beavis et al., 1994; Veldboom et al., 1994; Austin and Lee, 1996; Lima et al., 2006; Messmer et al., 2009). Combined with map-based cloning, QTL mapping has also been shown to be an efficient strategy to detect underlying genes and elements (Bommert et al., 2013). However, the high complexity of crop genomes and the low-coverage of genetic markers across chromosomes have posed great challenges for dissection of quantitative genetic variation by QTL analysis, especially for detecting small-effect QTL (Wenzl et al., 2006; Yu et al., 2011).

Along with the appearance of the first maize genetic linkage map in 1986 based on restriction fragment length polymorphisms (RFLP) technology (Helentjaris et al., 1986), molecular markers based on PCR technology, such as simple sequence repeats (SSRs) (Senior et al., 1996), expressed sequence tags (ESTs) (Davis et al., 1999), and amplified fragment length polymorphisms (AFLPs) (Vuylsteke et al., 1999) were further developed and applied in constructing maize genetic linkage maps. Subsequently, large number of QTLs for maize complex traits were detected and mapped on all 10 maize chromosomes based on these linkage maps (Tsonev et al., 2009; Qiu et al., 2011). However, low marker density on these maps limits QTL mapping accuracy, which leads to low QTL mapping resolution (Beavis et al., 1994; Veldboom et al., 1994). Along with the development in the next-generation sequencing (NGS) technologies and the continuous declining cost of genotyping, it is possible to develop high-quality SNP markers for genotyping of maize mapping populations. Genotyping-by-sequencing (GBS) (Elshire et al., 2011) is a popular new method for developing high density SNPs for constructing genetic linkage maps and has been successfully utilized for genetic studies in various species (Poland et al., 2012; Byrne et al., 2013; Sonah et al., 2013; Spindel et al., 2013), including maize (Chen et al., 2014; Zhou et al., 2016).

Association studies have also been successfully used for the genetic analysis of yield traits. To date, using different populations, more than 36 QTLs for traits related to cob diameter have been identified on all 10 maize chromosomes except chromosome 6 and most of these QTLs are located on chromosome 1 and 2 (Gramene QTL database). More than 45 QTLs for traits related to ear diameter have been identified on all maize chromosomes. More than 149 QTLs for traits related to 100-kernel weight have been identified. More than 46 QTLs for traits related to ear length have been identified on all maize chromosomes except chromosome 7. More than 23 QTLs for traits related to kernel row number have been identified on nine of the 10 maize chromosomes. More than 26 QTLs for traits related to grain number per panicle have been identified on six of the 10 maize chromosomes. A recent study of genome-wide dissection of the maize ear genetic architecture using multiple populations carried out by Xiao et al. (2016) showed that a total 243 QTLs for maize ear traits have been mapped. Genome-wide association studies (GWAS) were carried out for 17 agronomic traits, e.g., 100-grain weight, cob diameter and ear diameter, with a panel of 513 maize inbred lines and 343 significant loci were reported (Yang et al., 2014). A total 42 associated SNPs were identified, located in 33 genes for 126 trait × environment × treatment combinations (Austin and Lee, 1996).

Construction of large advanced crop populations can be both time consuming and expensive. In addition, Vales et al. (2005) concluded that early generation population is beneficial for detecting more QTLs, including small-effect QTLs (Vales et al., 2005). The purposes of this study were (1) to develop bin markers from high-throughput GBS data in a set of F_2_ individuals derived from two maize inbred lines SG-5 and SG-7; (2) to construct a high-density linkage map based on these bin markers; (3) to map QTLs for 100-kernel weight, ear length, ear diameter, cob diameter, kernel row number, corn grains per row, ear weight, and grain weight per plant in the F_2_ population, and to predict candidate genes for the detected QTLs with small physical intervals using maize gene annotations.

## Results

### Genome wide identification of SNPs using GBS

**Figure 1:**
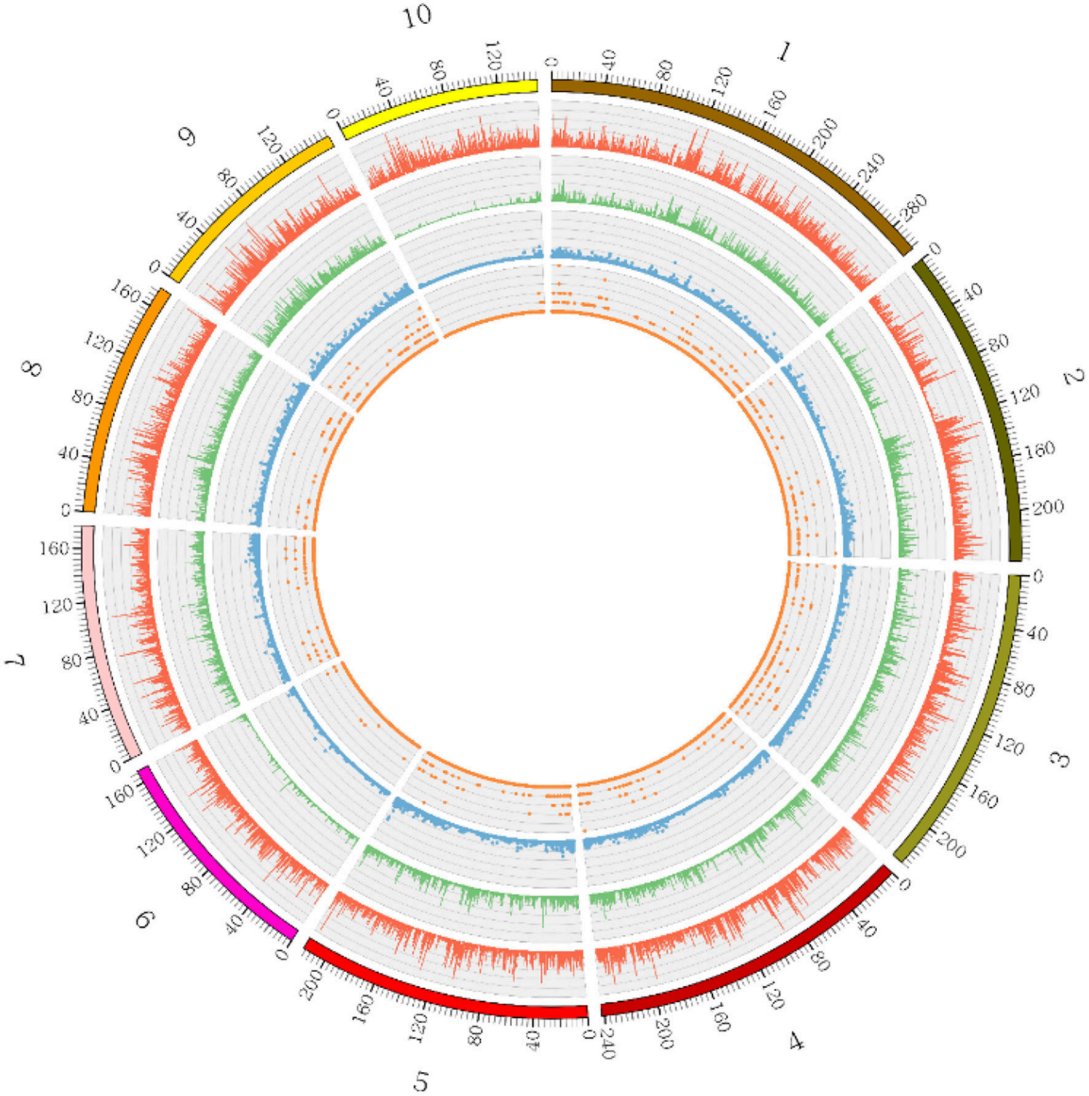
**Distribution and structural annotation of SNPs**. Distributions of SNPs detected on each maize chromosome (5 kb window size) are shown in the Circos diagram. Track 1 represents the 10 maize chromosomes (1–10) in different colors. Tracks 2, 3, 4, and 5 represent raw SNPs, filtered SNPs, genes and exons, respectively. Different tracks are represented by different colors as indicated.

**Figure 2:**
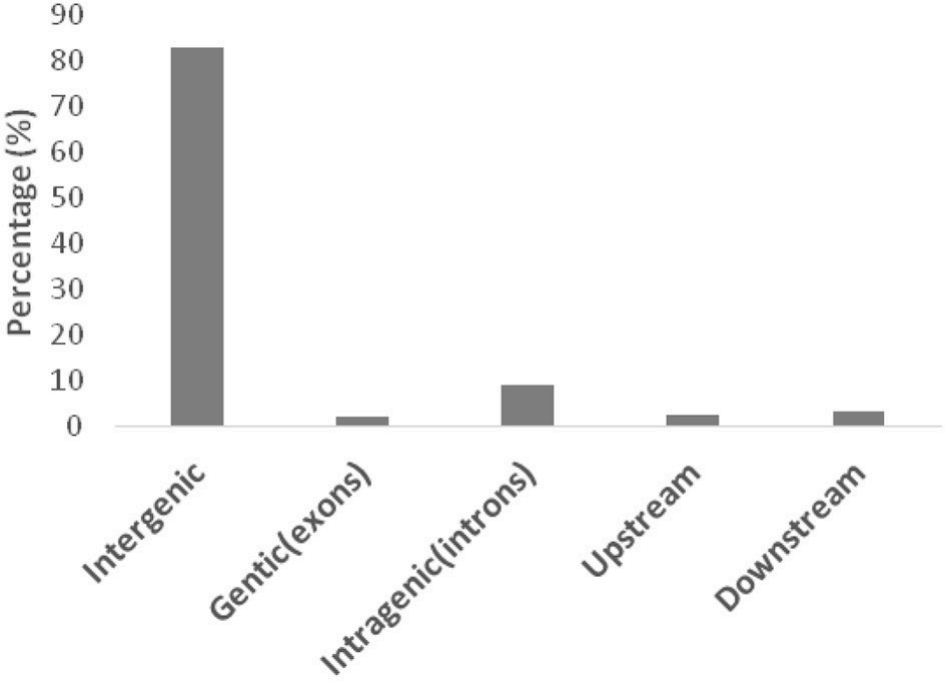
**Distribution of SNPs with the aa × bb segregation pattern on the basis of their locations in different genomic regions**.

For genome-wide detection of SNPs from maize using GBS, the restriction enzyme Mse I and Hae III were used to digest genomic DNA and construct GBS libraries of the F_2_ lines and the parents of the intra-specific mapping population (SG-5 and SG-7). Sequencing was carried out in an Illumina high-throughput sequencing platform Illumina Hiseq™ sequencer and a total of 1,059,026,818 reads were generated. A total of 1,046,524,604 high quality filtered reads successfully passed the QC steps as the remaining reads were filtered out due to the lack of proper layout of barcodes and restriction sites. The average number of reads per individual was 5,258,918 (Figure S1), which is equivalent to approximately 0.36-fold coverage of the maize genome. The overall GC content of the sequences was about 40.43%. Q20 and Q30 scores were about 96.37 and 91.34%, respectively. The 144-mer short reads of parents and F_2_ individuals were aligned with the Zea_mays.AGPv3.29 sequence to retrieve the physical position of each SNP. The SNPs were found to be distributed across all 10 maize chromosomes as illustrated in Figure 1 and Table S2. A total of 133,936 polymorphic SNPs between the two parental lines were identified by low-coverage sequencing (Table S1). Because the two parents are homozygous inbred lines with genotypes of aa and bb, only 68,882 homozygous polymorphic SNPs fell into the aa × bb segregation pattern (Figure 2 and Figure S2). The maize genome annotation project database (ftp://ftp.ensemblgenomes.org/pub/plants/release-29/fasta/zea_mays/dna/Zea_mays.AGPv3.29.dna.toplevel.fa.gz) was used to delineate the location of the GBS derived 68,882 SNPs in the genomic regions: intergenic, genic (exons), intragenic (introns), and UTRs. Majority (82.8%) of the SNPs were located in intergenic regions (Figure 2). Of the remaining, the largest numbers of SNPs were found to be located within introns (9.2%) followed by downstream (3.2%), upstream (2.6%), and exons (2.1%). In the F_2_ population, SNPs should segregate in a 1:2:1 ratio. SNPs exhibiting significant segregation distortion (*p* < 0.001, Chi-square test) were filtered out. Additionally, SNPs containing abnormal base and with more than 25% missing values across the genotyped individual were also deleted. Subsequently, a total of 29,927 SNPs were used to infer bins. A bin is defined as a perfect linkage disequilibrium (LD) block within which all SNPs segregate identically. The list of SNPs along with their flanking sequences are given in Table S3 (number per 100 kb). The number of raw and filtered SNPs and their frequencies (number per 100 kb) varied across chromosomes and closely mirrored the distribution of genes and exons. The largest number of raw SNPs were found on chromosome 1 (457,898). The highest frequency (154.78) of raw SNPs per 100 Kb occurred on chromosome 10 and lowest frequency (135.04) on chromosome 6. After filtering, chromosome has the maximum number of SNPs (4643).

### Genetic linkage map with bin markers

**Figure 3:**
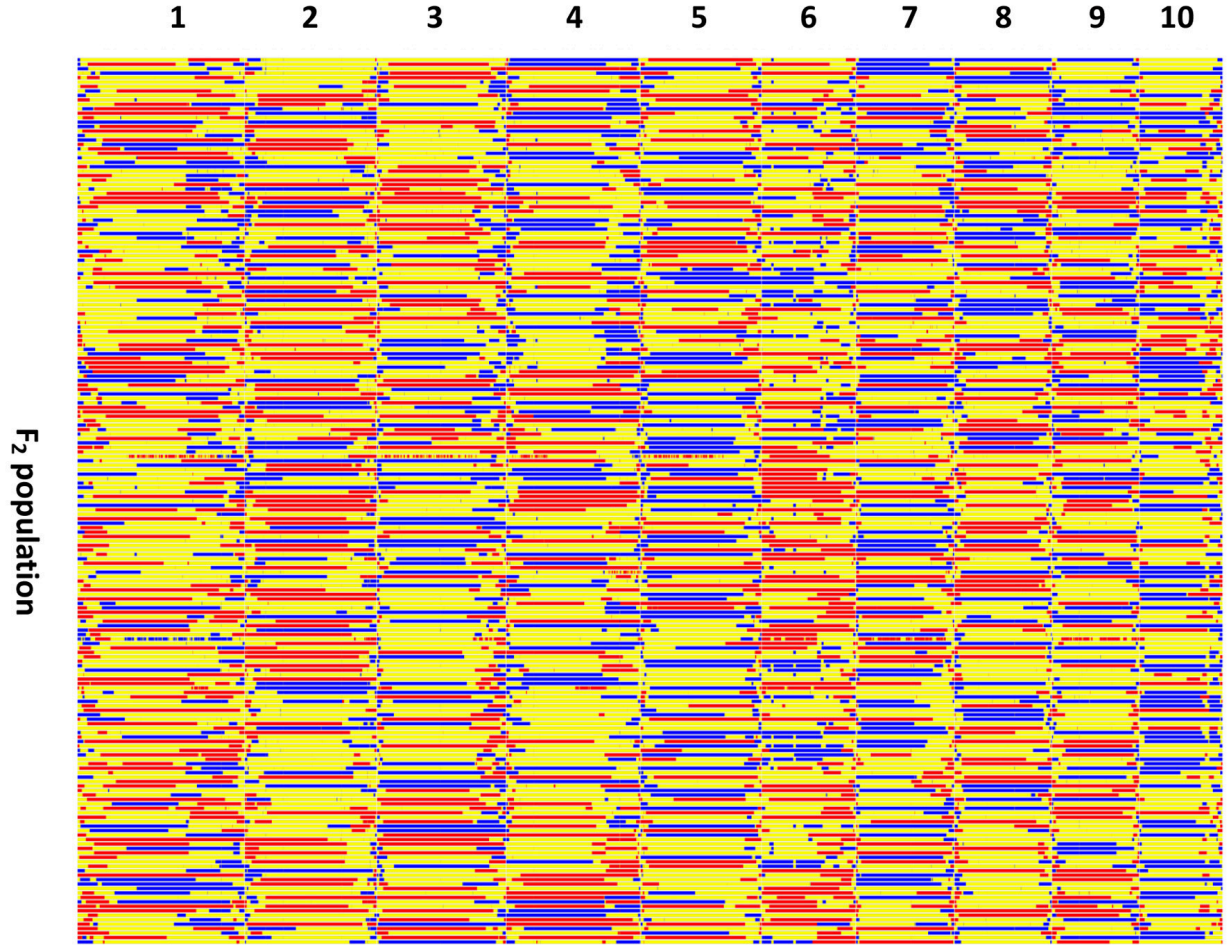
**Recombination bin-map of the F_**2**_ population**. Bin-map consists of 3,305 bin markers inferred from 29,927 high quality SNPs in the F_2_ population. Physical position is based on the Zea_mays.AGPv3.29 sequence. Red, SG5 genotype; Blue, SG7 genotype; Yellow, heterozygote.

The recombination maps were divided into skeleton bins (Huang et al., 2009) for further genetic analysis. A total of 3,305 bins were formed as described in the Method Section (Figure 3). The length of bin markers ranges from 50 Kb to 21.65 Mb, with a mean of 622.2 Kb, and a median of 350 Kb. In total, 71.5% of bin markers are less than 0.6 Mb in length. There are 255 bins larger than 1.5 Mb in size and six bins longer than 10.0 Mb dispersed on chromosomes 2 (mk746, mk749, and mk750), 4 (mk1562 and mk1563), and 10 (mk3222) (see Figure S3). A high-density genetic map was constructed by mapping these 3,305 bin markers onto the 10 maize chromosomes (Figure S4). The total length of the linkage map is 2236.66 cM with LG2 (382.80 cM) being the largest and LG10 (139.51 cM) being the smallest. The average distance between two adjacent markers is 0.68 cM. The number of markers per linkage group varies from 120 (LG10) to 623 (LG1), with an average of 330.5 markers per linkage group. The average marker density with LG1 having the highest marker density (0.497 cM per interval) and LG2 having the lowest density (1.190 cM per interval). Few gaps were observed, most of which are between 5 and 10 cM in length with the largest being 34.51 cM on LG2 (Table S4). A summary of the constructed genetic map is presented in Table 1.

**Table 1:** **Summary statistics of the maize intra-specific genetic linkage map constructed using F_**2**_ individuals derived from the cross of SG5 and SG7**.

**Chromosome**	**Number of bins**	**Chromosome size (cM)**	**Ave Gap (cM)**	**Max Gap (cM)**
1	623	309.26	0.50	12.32
2	324	382.80	1.19	34.51
3	450	228.78	0.51	9.92
4	415	279.34	0.67	20.83
5	381	189.79	0.50	5.98
6	141	153.86	1.10	7.65
7	291	185.30	0.64	13.30
8	253	167.92	0.67	5.18
9	307	200.09	0.65	13.23
10	120	139.51	1.17	13.53
Total	3,305	2,236.66	0.68	34.51

### Evaluation of phenotypic data

Phenotyping data were collected for 100-kernel weight (KW), ear length (EAL), ear diameter (EAD), cob diameter (CD), kernel row number (KRN), corn grains per row (CGR), ear weight (EW), and grain weight per ear (GWP) in 2014 for the F_2_ mapping population (*Zea mays* L. SG5 × *Zea mays* L. SG7). Significant differences for eight traits were observed within the F_2_ population and between the parental genotypes. Descriptive statistics of the traits analyzed in this study are summarized in Table 2. Bell shaped normal distribution was observed for all the traits analyzed (Figure S5). Pearson's correlation coefficients between the phenotypic traits are given in Table 3 along with their significance tests. The highest correlation occurs between GWP and EW (0.974).

**Table 2:** **Descriptive statistics of traits in the F_**2**_ mapping population of maize derived from the cross of SG5 and SG7**.

**Trait**	**SG5 (P1)**	**SG7 (P2)**	**Min**	**Max**	**Mean**	**Std. Dev.**
Ear length (cm)	14.38	12.72	9.10	26.00	14.49	2.27
Ear diameter (cm)	3.72	5.02	3.10	5.40	4.45	0.36
Cob diameter (cm)	2.53	3.24	2.10	3.90	2.94	0.28
Kernel row number	9.00	16.00	7.00	18.00	12.24	1.81
Grain number per row	23.23	17.23	7.33	33.67	24.44	4.86
Ear weight (g)	142.87	72.96	47.97	233.84	132.49	34.07
Grain weight per cob (g)	111.07	51.12	24.24	186.69	98.18	28.30
100-kernel weight (g)	34.79	26.41	20.00	44.36	34.09	4.39

**Table 3:** **Pearson correlations for yield related traits of maize from the F_**2**_ population of SG5 × SG7**.

**Trait**	**EAL**	**EAD**	**CD**	**KRN**	**CGP**	**EW**	**GWP**	**KW**
EAL	1							
EAD	0.080	1						
CD	0.033	0.572^**^	1					
KRN	−0.010	0.472^**^	0.339^**^	1				
CGR	0.618^**^	0.128	0.016	0.000	1			
EW	0.682^**^	0.461^**^	0.206^**^	0.238^**^	0.712^**^	1		
GWP	0.652^**^	0.433^**^	0.132	0.275^**^	0.720^**^	0.974^**^	1	
KW	0.352^**^	0.285^**^	0.114	−0.228^**^	0.094	0.384^**^	0.359^**^	1

** *Significantly different from 0 at alpha = 0.05*.

### Identification of QTLs

**Figure 4:**
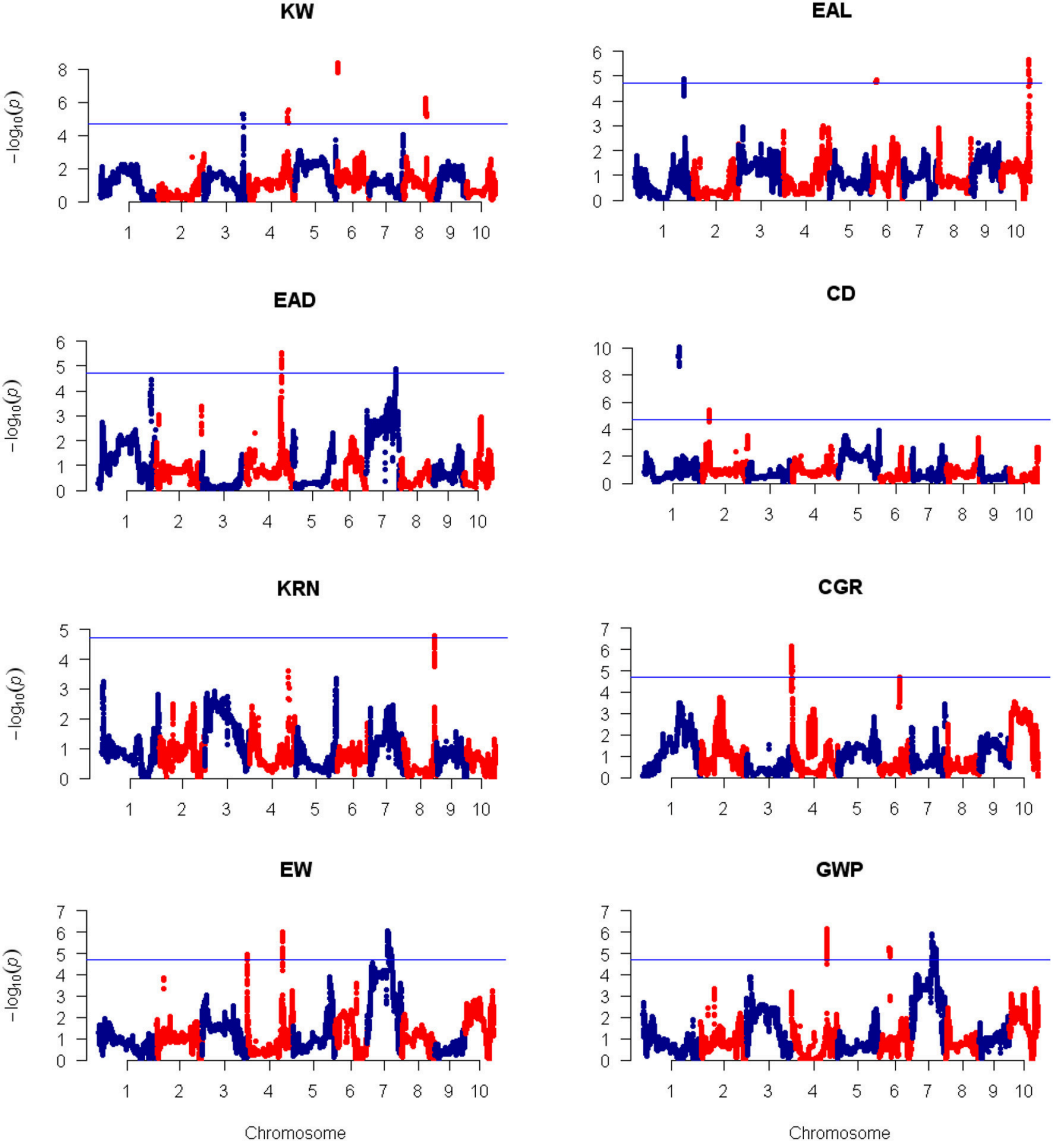
**Plots of test statistic −Log10(***p***) against genome location for eight traits of maize using the CIM method**. The horizontal blue line of each panel is the critical value of the test statistic generated from 1,000 permuted samples. The eight traits are: ear length (EAL), ear diameter (EAD), cob diameter (CD), kernel row number (KRN), corn grains per row (CGR), ear weight (EW), and grain yield per plant (GWP).

**Figure 5:**
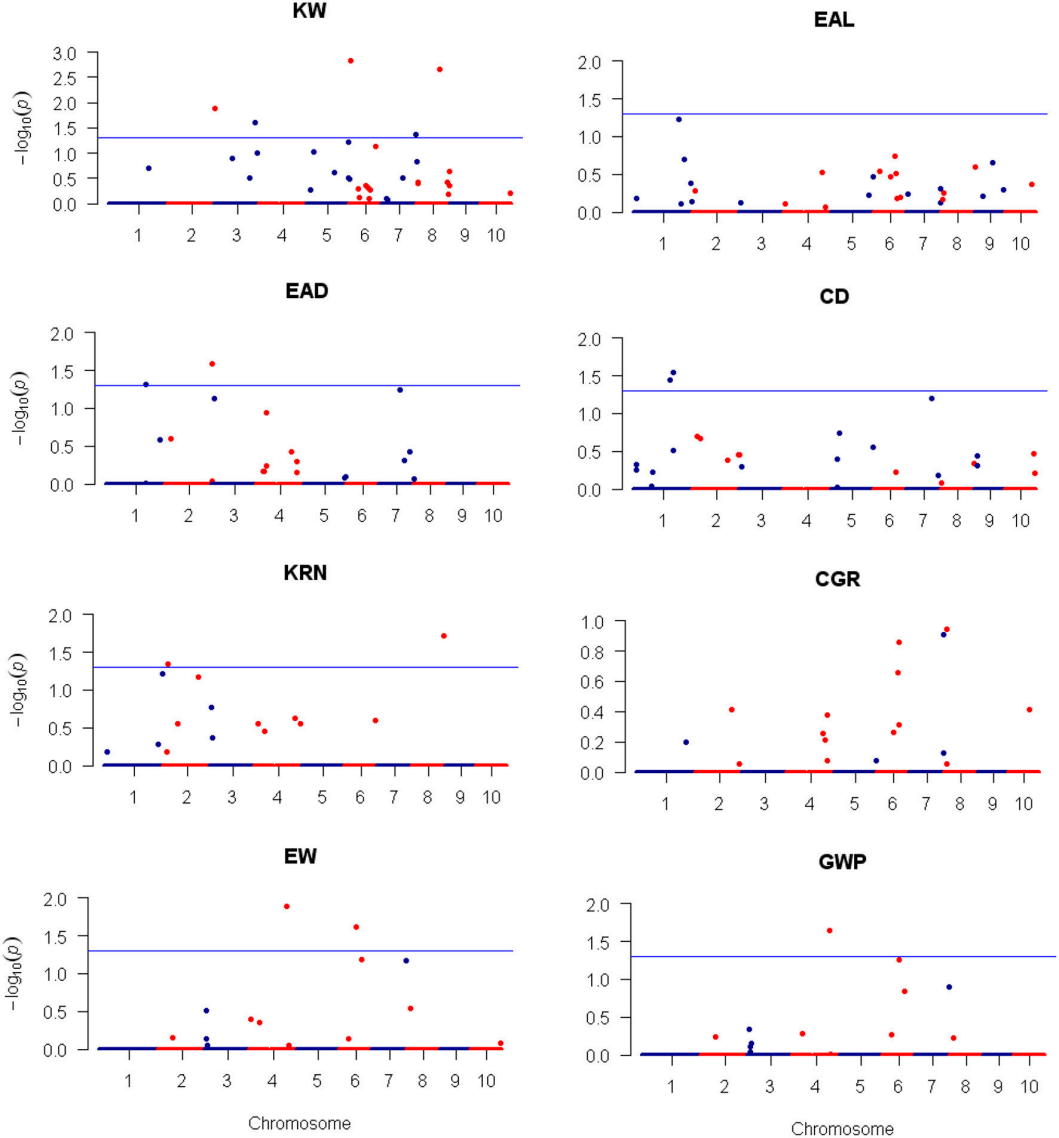
**Plots of the additive effect test statistic −Log10(***p***) against genome location for eight traits of maize using the LASSO method**. The horizontal blue line is the critical value of the test statistic at the nominal level of −Log10(0.05) = 1.3. Note that LASSO is a multiple marker model and no adjustment for the critical value of test statistic is required.

**Figure 6:**
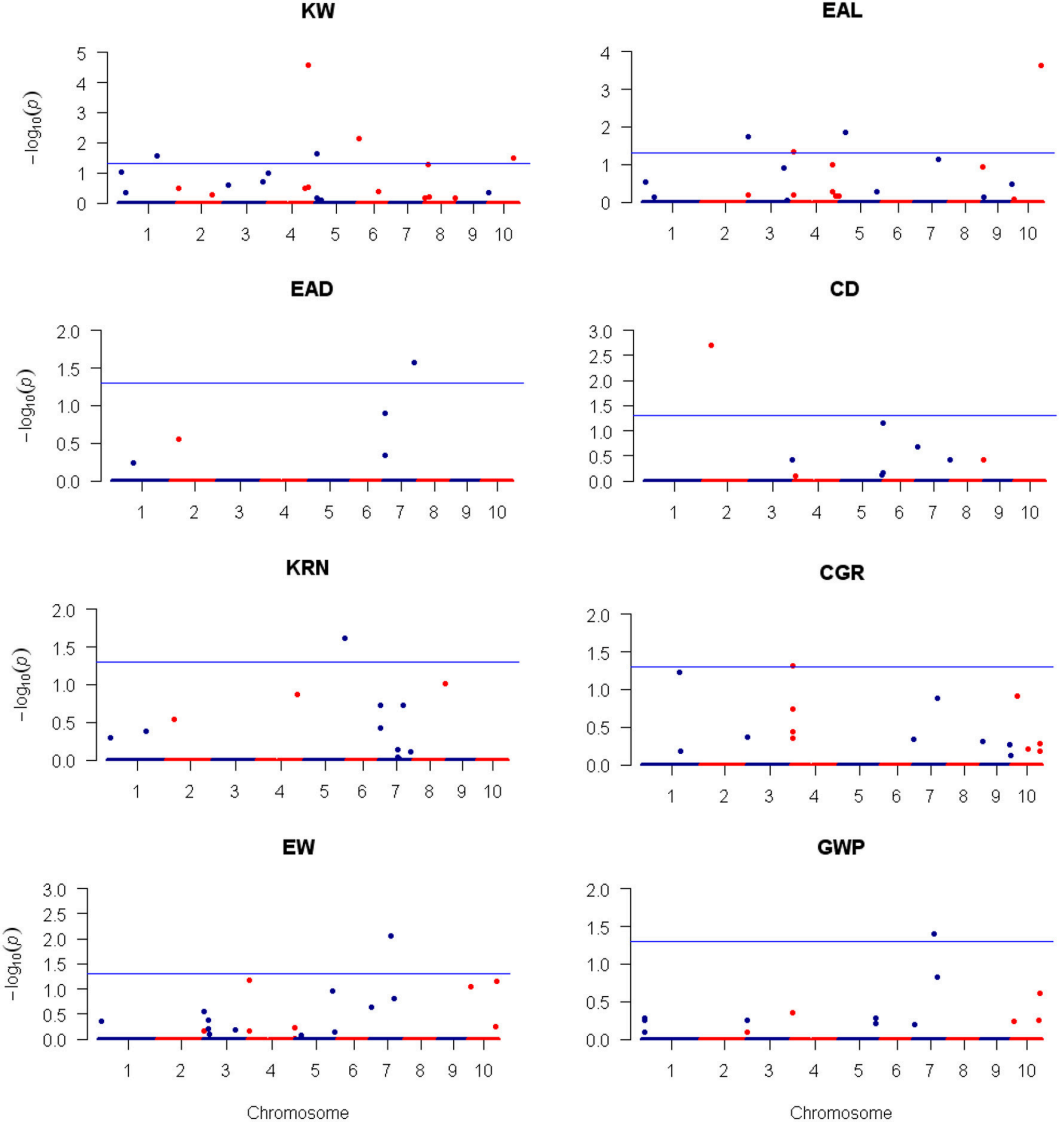
**Plots of the dominance effect test statistic −Log10(***p***) against genome location for eight traits of maize using the LASSO method**. The horizontal blue line is the critical value of the test statistic at the nominal level of −Log10(0.05) = 1.3. Note that LASSO is a multiple marker model and no adjustment for the critical value of test statistic is required.

Using the 3,305 bin-markers mapped on the intra-specific linkage map, we performed QTL mapping for the eight traits using the composite interval mapping (CIM) method and the LASSO method. Manhattan plot of the result is shown in Figure 4 for the CIM method. The corresponding plots for the LASSO method are shown in Figures 5, 6 for the additive effect test and dominance effect test, respectively. For CIM method, a total of 28 QTLs were identified for the following eight traits: EAL, EAD, CD, KRN, CGR, EW, GWP, and KW: four of them influence KW and are distributed on chromosomes 3, 4, 6, and 8; four of them influence EAL and are distributed on chromosomes 1, 6, and 10; five of them influence EAD and are distributed on chromosomes 1, 4, and 7; two of them influence CD and are distributed on chromosomes 1 and 2; one of them influences KRN and is located on chromosome 8; three of them influence CGR and are distributed on chromosomes 4 and 6; five of them influence EW and are distributed on chromosomes 4 and 7; five of them influence GWP and are distributed on chromosomes 4, 6, and 7. The confidence intervals for these 28 QTLs spanned physical distances from 0.2 to 40.7 Mb by comparison to the Zea_mays.AGPv3.29 genome. The phenotypic variation explained by each QTL ranged from 6.4 to 19.7% of the variation in a trait, with means of 9.4, 8.43, 8.48, 12.85, 7.6, 9.33, 11.28, and 9.68% for KW, EAL, EAD, CD, KRN, CGR, EW, and GWP, respectively. The identified QTLs are distributed on all the LGs except LG5 and LG9. The LOD scores range from 4.0 (*qEAD-4* and *qCGR-3*) to 9.1 (*qCD-1*). Information of the identified QTLs is summarized in Table 4.

**Table 4:** **QTL identified for nine traits of maize using high-density SNP bin-map from composite interval mapping (CIM)**.

**Trait**	**QTL**	**Chr**	**Flanking markers**	**Positions (Mb)**	**Interval (100 Kb)**	**Physical length (Mb)**	**LOD**	**ADD^a^**	**DOM^b^**	***R*^2^ (%)**	**QTL-MI^c^**	**References**
**PERMUTATION 1,000 TIMES WITH ALPHA** = **0.05**												
KW	*qKW-1*	Lg3	mk1315–mk1322	204.30	2,036.51–2,065.51	2.900	4.5	−1.54	1.23	7.7		
	*qKW-2*	Lg4	mk1708–mk1711	205.80	2,055.51–2,063.01	0.750	4.8	−0.16	2.43	7.4		
	*qKW-3*	Lg6	mk2201–mk2203	5.95	40.42–64.69	2.427	7.5	1.96	1.44	13		
	*qKW-4*	Lg8	mk2775–mk2777	119.70	1,194.01–1,206.01	1.200	5.5	−1.89	0.99	9.5		
EAL	*qEAL-1*	Lg1	mk478–mk492	254.45	2,493.51–2,554.51	6.100	4.1	−0.84	−0.44	8.2		
	*qEAL-2*	Lg6	mk2219–mk2227	27.45	198.01–363.01	16.500	4.1	0.99	−0.54	8.1		
	*qEAL-3*	Lg10	mk3282–mk3292	145.20	1,440.51–1,466.51	2.600	4.9	−0.33	1.24	9		
EAD	*qEAD-1*	Lg1	mk331–mk339	196.50	1,963.01–1,981.01	1.800	4.7	0.14	0.06	8.3	184.9–187.4	Xue et al., 2013
	*qEAD-2*	Lg4	mk1648–mk1652	185.35	1,839.51–1,855.51	1.600	5.3	0.13	0.13	9.2	190.78–217.64	Beavis et al., 1994
	*qEAD-3*	Lg7	mk2348–mk2353	5.15	47.01–63.51	1.650	4.3	0.09	0.17	6.9		
	*qEAD-4*	Lg7	mk2468–mk2471	108.30	1,073.01–1,089.01	1.600	4.0	0.11	0.17	6.4		
	*qEAD-5*	Lg7	mk2541–mk2544	154.00	1,537.01–1,552.51	1.550	7.0	0.12	0.19	11.6	147.16	Xue et al., 2013
											143.17–161.8	Austin and Lee, 1996
CD	*qCD-1*	Lg1	mk298–mk303	180.20	1,781.01–1,823.01	4.200	9.1	0.17	0.06	18	187.4	Xue et al., 2013
	*qCD-2*	Lg2	mk700–mk708	36.95	333.51–371.51	3.800	4.6	0.06	0.12	7.7		
KRN	*qKRN-1*	Lg8	mk2821–mk2830	160.65	1,588.01–1,635.51	4.750	4.1	0.68	−0.39	7.6	163.5–164.6	Xiao et al., 2016
CGR	*qCGR-1*	Lg4	mk1407–mk1408	2.80	27.01–29.01	0.200	5.4	0.80	3.04	10		
	*qCGR-2*	Lg4	mk1414–mk1420	4.95	38.51–67.51	2.900	5.3	0.44	3.07	9.8		
	*qCGR-3*	Lg6	mk2267–mk2269	106.25	1,057.05–1,072.51	1.546	4.0	1.97	−0.10	8.2	106.47	Yang et al., 2014
EW	*qEW-1*	Lg4	mk1399–mk1419	3.25	3.01–61.01	5.800	4.2	9.53	15.53	8.4	3.7-4.7	Xiao et al., 2016
	*qEW-2*	Lg4	mk1650–mk1660	185.55	1,850.51–1,876.51	2.600	5.2	13.29	12.19	10.4	186.1–187.4	Xiao et al., 2016
	*qEW-3*	Lg7	mk2462–mk2467	106.00	1,052.51–1,073.01	2.050	5.2	51.91	18.99	19.7		
	*qEW-4*	Lg7	mk2468–mk2473	107.95	1,073.01–1,110.01	3.700	5.1	8.19	20.05	9.7		
	*qEW-5*	Lg7	mk2483–mk2484	122.30	1,216.51–1,232.01	1.550	4.4	−8.80	19.78	8.2		
GWP	*qGWP-1*	Lg4	mk1648–mk1659	185.55	1,839.51–1,874.51	3.500	5.3	12.35	6.75	10.7	186.1–187.4	Xiao et al., 2016
	*qGWP-2*	Lg6	mk2220–mk2240	52.95	207.01–614.01	40.700	4.4	14.33	−4.68	11.8		
	*qGWP-3*	Lg7	mk2462–mk2474	106.00	1,052.51–1,135.51	8.300	5.1	38.77	15.88	9.2		
	*qGWP-4*	Lg7	mk2468–mk2478	107.95	1,073.01–1,083.01	1.000	4.7	3.40	16.53	8.6	107.4–108.2	Xiao et al., 2016
	*qGWP-5*	Lg7	mk2485–mk2489	124.05	1,232.01–1,265.51	3.350	4.5	−5.63	16.36	8.1		

a *Estimated additive effect*.

b *Estimated dominance effect*.

c *Marker interval of QTLs identified in previous studies*.

For LASSO method, a total of 29 QTLs were identified for the eight traits: ten of them influence KW and are distributed over all chromosomes except 9; four of them influence EAL and are distributed on chromosomes 3, 4, 5, and 10; three of them influence EAD and are distributed on chromosomes 1, 2, and 7; three of them influence CD and are distributed on chromosomes 1 and 2; three of them influence KRN and are distributed on chromosomes 2, 5, and 8; one of them influences CGR and is located on chromosome 4; three of them influence EW and are distributed on chromosomes 4, 6, and 7; two of them influence GWP and are distributed on chromosomes 4 and 7. The −Log10(*p*)-value ranges from 1.31 (*qEAD-1* and *qCGR-2*) to 4.57 (*qKW-2*). Information of the identified QTLs is summarized in Table 5. These QTL are distributed on all the chromosomes except LG9 (Table 5). Among the QTLs identified, 16 of them were detected by both CIM and LASSO (see Table 4).

**Table 5:** **QTL identified for nine traits of maize using high-density SNP bin-map from the LASSO method**.

**Trait**	**QTL**	**Chr**	**Flanking marker**	**Positions (Mb)**	**Add/Dom**	**Effect**	**–Log10(*p*)**	**CIM^a^**	**QTL-MI**	**References**
KW	*qKW-6*	2	mk945	235.75	A	−0.86	1.88			
	*qKW-1*	3	mk1316	204.35	A	−1.73	1.60	Yes		
	*qKW-3*	6	mk2202	5.6	A	2.03	2.83	Yes		
	*qKW-7*	7	mk2622	174.9	A	−1.69	1.38			
	*qKW-4*	8	mk2776	119.75	A	−1.95	2.66	Yes		
	*qKW-8*	1	mk322	193.55	D	1.33	1.55		208.09	Xue et al., 2013
	*qKW-2*	4	mk1709	205.85	D	2.24	4.57	Yes		
	*qKW-9*	5	mk1844	7.15	D	1.60	1.64			
	*qKW-5*	6	mk2203	6.25	D	2.29	2.14	Yes		
	*qKW-10*	10	mk3249	124.8	D	1.54	1.49		124.73/125.11/125.9	Xue et al., 2013
EAL	*qEAL-4*	3	mk963	2.15	D	−0.92	1.73		2.6–2.8	Xiao et al., 2016
	*qEAL-5*	4	mk1413	3.7	D	0.93	1.35			
	*qEAL-6*	5	mk1903	30.2	D	1.00	1.86			
	*qEAL-3*	10	mk3287	145.75	D	1.15	3.64	Yes		
EAD	*qEAD-1*	1	mk334	196.85	A	0.14	1.31	Yes	184.9–187.4	Xue et al., 2013
	*qEAD-6*	2	mk945	235.75	A	−0.20	1.59			
	*qEAD-5*	7	mk2542	154.1	D	0.23	1.58	Yes	147.16	Xue et al., 2013
CD	*qCD-1*	1	mk300	180.55	A	0.17	1.45	Yes	187.4	Xue et al., 2013
	*qCD-3*	1	mk336	197.2	A	−0.17	1.54		187.4	Xue et al., 2013
	*qCD-2*	2	mk707	37.05	D	0.17	2.70	Yes		
KRN	*qKRN-2*	2	mk680	22.65	A	0.83	1.35		18.5–18.6	Xiao et al., 2016
	*qKRN-1*	8	mk2826	160.7	A	0.70	1.72	Yes	163.9–164.1	Xiao et al., 2016
	*qKRN-3*	5	mk2162	210.3	D	0.70	1.62		213.1–213.3	Xiao et al., 2016
CGR	*qCGR-2*	4	mk1417	5	D	2.72	1.31	Yes		
EW	*qEW-2*	4	mk1653	185.6	A	13.72	1.89	Yes	186.1–187.4	Xiao et al., 2016
	*qEW-6*	6	mk2254	85.7	A	14.91	1.62			
	*qEW-4*	7	mk2470	108.05	D	20.46	2.06	Yes		
GWP	*qGWP-1*	4	mk1653	185.6	A	12.14	1.65	Yes	186.1–187.4	Xiao et al., 2016
	*qGWP-4*	7	mk2470	108.05	D	16.86	1.40	Yes	107.4–108.2	Xiao et al., 2016

a *Indicates whether or not the QTL has been identified by the CIM method*.

### Candidate gene prediction

The small physical intervals of *qCGR-1, qKW-2*, and *qGWP-4* encompass 14, 24, and 20 protein coding genes, respectively (see Table 4 and Table S5), according to the maize gene annotation database accessible at MaizeGDB (http://www.maizegdb.org). Recent work in *Arabidopsis* and rice have shown that the F-box protein coding gene possibly regulates multiple aspects of flower development and leads to increased grain number (Ni et al., 2004; Ikeda et al., 2007). In addition, studies in *Arabidopsis* showed that WD40-repeat protein gene possibly plays an important role during the mitosis process of pollen nucleus and may regulate seed mass and seed size (You et al., 2011). Red color 1 (Pilu et al., 2012), involved in regulating anthocyanin pigmentation in different maize tissues, is also an enhancing gene for ear weight and plant height. UDP-Glycosyltransferase genes have been proven to be the key genes regulating anthocyanin biosynthesis in grape (Boss et al., 1996a,b; Kobayashi et al., 2001). Among the candidate genes in the intervals of *qCGR-1*, one gene (GRMZM2G139872) is an F-box family protein gene. Among the candidate genes in the intervals of *qKW-2*, three genes (GRMZM2G180811, GRMZM5G828139, and GRMZM5G873194) are the WD40-repeat protein genes. Of the candidate genes in the intervals of *qGWP-4*, one gene (GRMZM2G019183) is the UDP-Glycosyltransferase gene.

## Discussion

The GBS technology is able to produce genotypes of a large number of markers with potentially less ascertainment bias than standard single nucleotide polymorphism (SNP) arrays (Crossa et al., 2013). The technology represents a novel application of the NGS protocol for detecting and genotyping SNPs in fields of crop improvement (He et al., 2014). It is a simple highly multiplexed system for constructing reduced representation libraries for the Illumina NGS platform developed in the Buckler lab (Elshire et al., 2011). Large numbers of SNPs can be identified for genotyping and genetic analyses (Beissinger et al., 2013). Key features of this system include low cost, reduced samples, fewer PCR and purification steps, no size fractionation, no reference sequence limits, efficient barcoding, and easiness to scale up (Davey et al., 2011). The low cost of GBS makes it an attractive approach to saturating a mapping or breeding population with high density of SNP markers. GBS has become a cost-competitive alternative to other whole genome genotyping platforms. As an ultimate marker assisted selection (MAS) tool and a cost-effective technique, GBS has been successfully applied to genome-wide association study (GWAS), genomic diversity study, genetic linkage analysis, molecular marker discovery, and genomic selection under large scales of plant breeding programs (He et al., 2014). The bin-map strategy has proven to be efficient in generating ultrahigh density of bin markers and detecting QTLs with high resolution in crop species (Yu et al., 2011; Zou et al., 2012; Xu, 2013). Compared with conventional molecular markers such as RFLP/SSR and single SNP markers, bin markers are the most informative, and parsimonious set for a given population (Chen et al., 2014). In the current study, we reported such a large-scale SNP discovery by GBS with low cost and simultaneous genotyping of F_2_ of an intra-specific mapping population of maize. The SNP calling and imputation processes were conducted by comparing the clean reads generated by GBS and reference genome Zea_mays.AGPv3.29 information. In one of our previous studies, a genetic map with 250 SSR markers was constructed based on 114 BC1F1 plants in a soybean intra-specific backcross population (Su et al., 2010). The average genetic distance between two adjacent markers was 11.85 cM, corresponding to a physical distance of about 4.4 Mb. In this study, a genetic map was generated by mapping 3,305 bin markers which consist of 29,927 filtered SNPs onto the 10 maize chromosomes. The length of bin markers ranged from 50 Kb to 21.65 Mb, with a mean of 622.2 Kb, and a median of 350 Kb. In total, 71.5% of the bin markers are less than 0.6 Mb in length. The average distance between two adjacent bin-markers is 0.68 cM, corresponding to a physical distance of about 0.69 Mb. We have shown that the identified QTLs can be narrowed down to relative small physical intervals of the target genome.

Results of QTL mapping depend on many factors, e.g., type of population, characteristics of traits, sample size, marker density, QTL mapping procedure, and so on. Understanding these factors can help investigators choose an optimal design of experiment and an optimal procedure for data analysis. For example, QTL for low heritability traits are often hard to detect. QTL for highly polygenic traits are also hard to detect, even if the traits may be highly heritable. Results from Olakojo and Olaoye (2011) showed that the heritability of kernel row number and grain yield in maize were only 5.7 and 16.22%, respectively. QTL mapping for these traits may be very hard. The genetic background also affects the power of QTL detection. A polygenic trait may have a very heterogeneous genetic background. Without proper control of the heterogeneous background, statistical power of QTL detection can be very low (Gallais, 2003). In one of our previous studies, a small-effect QTL *Flwdt7* conferring flowering time of soybean was mapped on LG C2 and it only contributed 11.0% of the phenotypic variance in the BC1F3 genetic background while in the advanced residual heterozygous line (RHL) populations the contribution increased to 36.8% (Su et al., 2010). In present study, we used two QTL mapping methods procedures, CIM and LASSO. The CIM method is an ad hoc method because the model only tests one marker at a time, although multiple loci are included in the model but they are used to control the background. One attractive feature of CIM is that it can handle markers with extremely high density. With 3,305 bin-markers in this study, LASSO can easily handle this many markers. Therefore, we also analyzed the data using the LASSO method. Because LASSO deals with a multiple marker model where all marker effects are estimated simultaneously, the result should be more reliable than the CIM method. The only limitation of LASSO is the inefficiency of handling extremely large number of markers. When the number of markers reaches more than 100,000 and the sample size is relatively small, the program tends to fail (Hu et al., 2012).

In the current study, 29 QTL have been detected with LASSO and 28 detected with CIM. Of these detected QTL, 16 of them overlapped (detected by both methods). The two methods seem to be quite consistent. All the detected QTLs may be used in the future for follow up studies. Recently, Xiao et al. (2016) conducted a GWAS for genetic architecture of ear in multiple advanced generations of maize. They detected 243 QTLs for maize ear traits and these QTL are distributed overall all 10 chromosomes. Another GWAS of maize was carried out by Yang et al. (2014) for 17 agronomic traits with a panel of 513 maize inbred lines. A total of 343 significant loci were detected for the 17 traits. Compared with these results, the majority of QTL identified in this study (*qKW-8, qKW-10, qEAL-4, qEAD-1, qEAD-2, qEAD-5, qCD-1, qCD-3, qKRN-1, qKRN-2, qKRN-3, qCGR-3, qEW-1, qEW-2, qGWP-3*, and *qGWP-1*) are either overlapping with the QTLs detected by Xiao et al. (2016) and Yang et al. (2014) or in the vicinity of those QTLs (see Tables 4, 5).

## Conclusion

In this study, an ultra-high density genetic map of maize was constructed based on markers identified with the GBS technology from an intra-specific F_2_ population of maize. The results revealed a higher degree of synteny between SNPs identified here and the reference genome. This implies that this map is accurate enough for efficient QTL mapping. QTLs conferring eight yield traits of maize were identified based on this genetic linkage map. A total of five candidate genes of *qCGR-1* (one gene), *qKW-2* (three genes), and *qGWP-4* (one gene) were successfully predicted. The work will not only help to understand the genetic mechanisms of how yield traits are controlled, but also provide a basis for marker-assisted selection and map-based cloning in further studies.

## Materials and methods

### DNA extraction

A segregating population of 199 F_2_ plants derived from an intra-specific cross between *Zea mays* L. SG5 and *Zea mays* L. SG7 was grown in November 2014 at the Panxian Maize Breeding Station in Guizhou, China. Young healthy leaves from the two parents and each of the 199 F_2_ individuals were collected and frozen in liquid nitrogen, and then transferred to a −80°C freezer. Genomic DNA from the F_2_ population and parents were extracted following the manufacturer's protocols with the Plant Genomic DNA Kit (TIANGEN, Beijing, China). DNA degradation and contamination were monitored on 1% agarose gels. DNA purity was checked using the NanoPhotometer® spectrophotometer (IMPLEN, CA, USA). DNA concentration was measured using Qubit® DNA Assay Kit in Qubit® 2.0 Flurometer (Life Technologies, CA, USA).

### Genotyping by sequencing

Genotyping-by-sequencing (GBS) is an efficient method of high-throughput genotyping, which is based on RRL and high-throughput sequencing. First, we performed a GBS pre-design experiment. The enzymes and sizes of restriction fragments were evaluated using training data. Three criteria were considered: (i) the number of tags must be suitable for the specific needs of the research project; (ii) the enzymatic tags must be evenly distributed through the sequences to be examined; (iii) repeated tags must be avoided. These considerations improved the efficiency of GBS. Next, we constructed the GBS library in accordance to the pre-designed scheme. Genomic DNAs from each of the F_2_ individuals and the parents were incubated at 37°C with *MseI* (New England Biolabs, NEB), T4 DNA ligase (NEB), ATP (NEB), and *MseI* Y-adapter N containing barcode. Restriction-ligation reactions were heat-inactivated at 65°C, and then digested for additional restriction enzyme *HaeIII* (NEB) at 37°C. The restriction ligation samples were purified with Agencourt AMPure XP (Beckman). The PCR amplifications were performed using purified samples and Phusion Master Mix (NEB) in a single tube after adding universal primer and index primer to each sample. The PCR productions were purified and pooled using Agencourt AMPure XP (Beckman) and then run out on a 2% agarose gel. Fragments with 400–425 bp (with indexes and adaptors) in size were isolated using a Gel Extraction Kit (Qiagen, Valencia, CA). These fragment products were then purified using Agencourt AMPure XP (Beckman) and further diluted for sequencing. Finally, the 150-bp pair-end reads with insert sizes of 265–290 bp sequencing were performed upon the selected tags using an Illumina high-throughput sequencing platform Illumina Hiseq™ by the Novogene Bioinformatics Institute, Beijing, China. SNP genotyping and evaluation were then performed.

### Sequence data grouping and SNP identification

The sequences of each F_2_ individuals were sorted according to the barcodes. To make sure that reads are reliable and without artificial bias (low quality paired reads, which mainly resulted from base-calling duplicates and adapter contamination) in the following analyses, raw data (raw reads) of fastq format were first processed through a series of quality control (QC) procedures in-house C scripts. The QC standards were: (1) removing reads with ≥10% unidentified nucleotides (N); (2) removing reads with >50% bases having phred quality < 5; (3) removing reads with >10 nt aligned to the adapter, allowing ≤ 10% mismatches; (4) removing reads that contain the *HaeIII* sequence.

Burrows-Wheeler Aligner (BWA) (Li and Durbin, 2009) was used to align the clean reads of each F_2_ individual against the reference genome with settings “mem -t 4 -k 32 -M -R,” where -t is the number of threads, -k is the minimum seed length, -M is an option used to mark shorter split alignment hits as secondary alignments, and -R is the read group header line. Alignment files were converted to BAM files using the sort setting in the SAMtools software (Li et al., 2009). We only kept the pair with the highest mapping quality if multiple read pairs have identical external coordinates. Variants calling were performed for all samples by using the SAMtools software. SNPs were filtered by the Perl script. The software tool ANNOVAR (Wang et al., 2010) was used to annotate SNPs based on the GFF3 files from the Zea_mays.AGPv3.29 sequence (ftp://ftp.ensemblgenomes.org/pub/plants/release-29/fasta/zea_mays/dna/Zea_mays.AGPv3.29.dna.toplevel.fa.gz). Polymorphic markers between the parents were classified into eight segregation patterns, such as ab × cd, ef × eg, hk × hk, lm × ll, nn × np, aa × bb, ab × cc, and cc × ab, but only the aa × bb type between the parents was chosen as the parents of the F_2_ population.

### Bin map construction

Chi-square (χ^2^) tests were conducted for all SNPs to detect segregation distortion. Markers with segregation distortion test *p* < 0.001 or containing abnormal base were filtered out. All markers were deleted if there were more than 25% individuals with missing genotypes. A sliding-window approach was applied for variant calling errors and to calculate the ratio of SNP alleles derived from the two parental lines, SG-5 and SG-7 (Huang et al., 2009). Genotypic data were scanned with a window size of 15 SNPs and a step size of 1. For each individual, the ratio of SNP alleles from the two parental lines within the window was calculated. Windows with 11 or more SNPs from either parent were considered to be homozygous, while those with less SNPs from a single parent were considered heterozygous. Adjacent windows with the same genotypes were combined into a single block, whereas adjacent blocks with different genotypes were assumed to be at or near a recombination breakpoint. A bin marker was designated when consecutive 100-Kb intervals lacked a recombination event in the entire population. For construction of the linkage map, the genetic distance between bin markers was calculated using the Kosambi mapping function implemented in the *est.map* function of the R/qtl package (Broman et al., 2003). A Perl SVG module was used to generate the linkage map.

### Plant materials and phenotyping

The F_2_ population of 199 individuals was derived from the cross of maize inbred lines SG5 and SG7. The 100-seed weight of the two parents are 34.79 and 26.41 g, respectively, for SG5 and SG7. Detailed information of the trait measurements for the parents are listed in Table 3. Phenotypic data for 100-kernel weight, ear length, ear diameter, cob diameter, kernel row number, corn grains per row, ear weight, and grain weight per plant were collected from the F_2_ individuals. Plants grown in a field trial in 2014 at the Panxian of Guizhou Maize Breeding Station, Hainan, China.

### QTL analysis

QTL analysis was performed using two methods: (1) CIM implemented with QTL Cartographer v2.5 using the stepwise regression for co-factor selection; (2) least absolute shrinkage and selection operator (LASSO) method implemented with the GLMNET/R software package (citation). For the CIM method, the LOD score threshold was determined by the result of 1,000 permutations for each trait. The software also estimated the percentage of phenotypic variance, additive effect and dominance effect explained by a QTL for a trait. For the LASSO method, the *p* = 0.05 was used as the threshold of the *p*-value, which translates into −log10(*p*) = 1.3 in this scale. The reason for not using permutation test for the LASSO method is that it is a multiple regression model with severe shrinkage on each marker effect. The nominal level of 0.05 applies to multiple regression analysis (Hu et al., 2012).

## Author contributions

CS and WW have finished phenotyping and genotyping of F2 progeny, participated in developing the F2 population, and statistical analysis of data. SG, JZ, and SL have provided advices on designing experiment. SX have finished analyzing the data, evaluating the QTL mapping methods, and drafted the manuscript. All authors read and approved the final manuscript.

### Conflict of interest statement

The authors declare that the research was conducted in the absence of any commercial or financial relationships that could be construed as a potential conflict of interest.
