# Supplementary material for: High Density Linkage Map Construction and Mapping of Yield Trait QTLs in Maize (Zea mays) Using the Genotyping-by-Sequencing (GBS) Technology
Source: Front Plant Sci. 2017 May 8;8:706. doi: 10.3389/fpls.2017.00706 (PMC5420586; doi:10.3389/fpls.2017.00706)
Supplement: Supplementary file 1 [file DataSheet1.DOCX]

a

Reads (in million)

b

Call rate (in percentage)

**Figure S1.** Number of reads in individual samples. a: Number of reads (in million); b: Call rate (in percentage).
