# Supplementary material for: High Density Linkage Map Construction and Mapping of Yield Trait QTLs in Maize (Zea mays) Using the Genotyping-by-Sequencing (GBS) Technology
Source: Front Plant Sci. 2017 May 8;8:706. doi: 10.3389/fpls.2017.00706 (PMC5420586; doi:10.3389/fpls.2017.00706)
Supplement: Supplementary file 2 [file DataSheet2.DOCX]

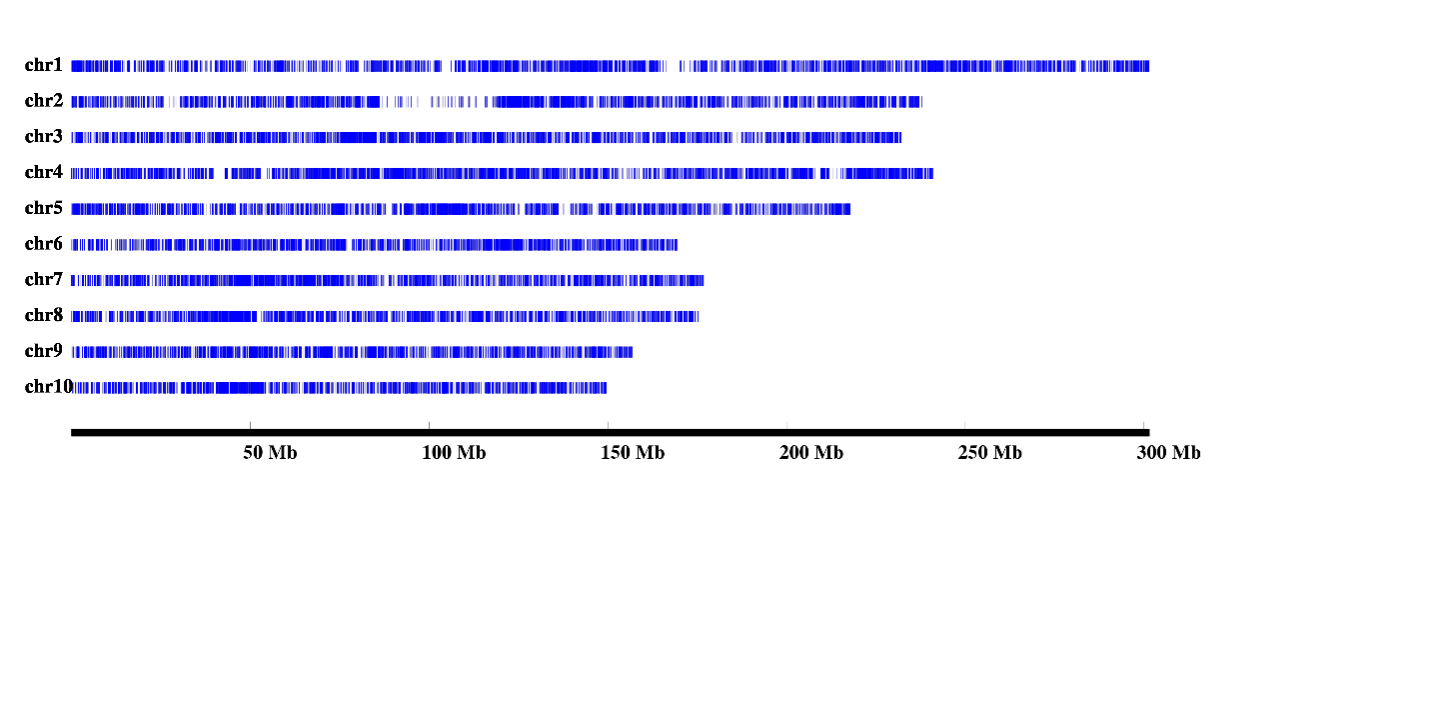


**Figure S2.** The distribution of SNPs with the aa×bb segregation pattern of over 10 chromosomes of maize.
