# Supplementary material for: High Density Linkage Map Construction and Mapping of Yield Trait QTLs in Maize (Zea mays) Using the Genotyping-by-Sequencing (GBS) Technology
Source: Front Plant Sci. 2017 May 8;8:706. doi: 10.3389/fpls.2017.00706 (PMC5420586; doi:10.3389/fpls.2017.00706)
Supplement: Supplementary file 3 [file DataSheet3.DOCX]

Frequency

Length of bins (Kb)

**Figure S3.** The distribution of bin marker length.
