# Supplementary material for: High Density Linkage Map Construction and Mapping of Yield Trait QTLs in Maize (Zea mays) Using the Genotyping-by-Sequencing (GBS) Technology
Source: Front Plant Sci. 2017 May 8;8:706. doi: 10.3389/fpls.2017.00706 (PMC5420586; doi:10.3389/fpls.2017.00706)
Supplement: Supplementary file 4 [file DataSheet4.DOCX]

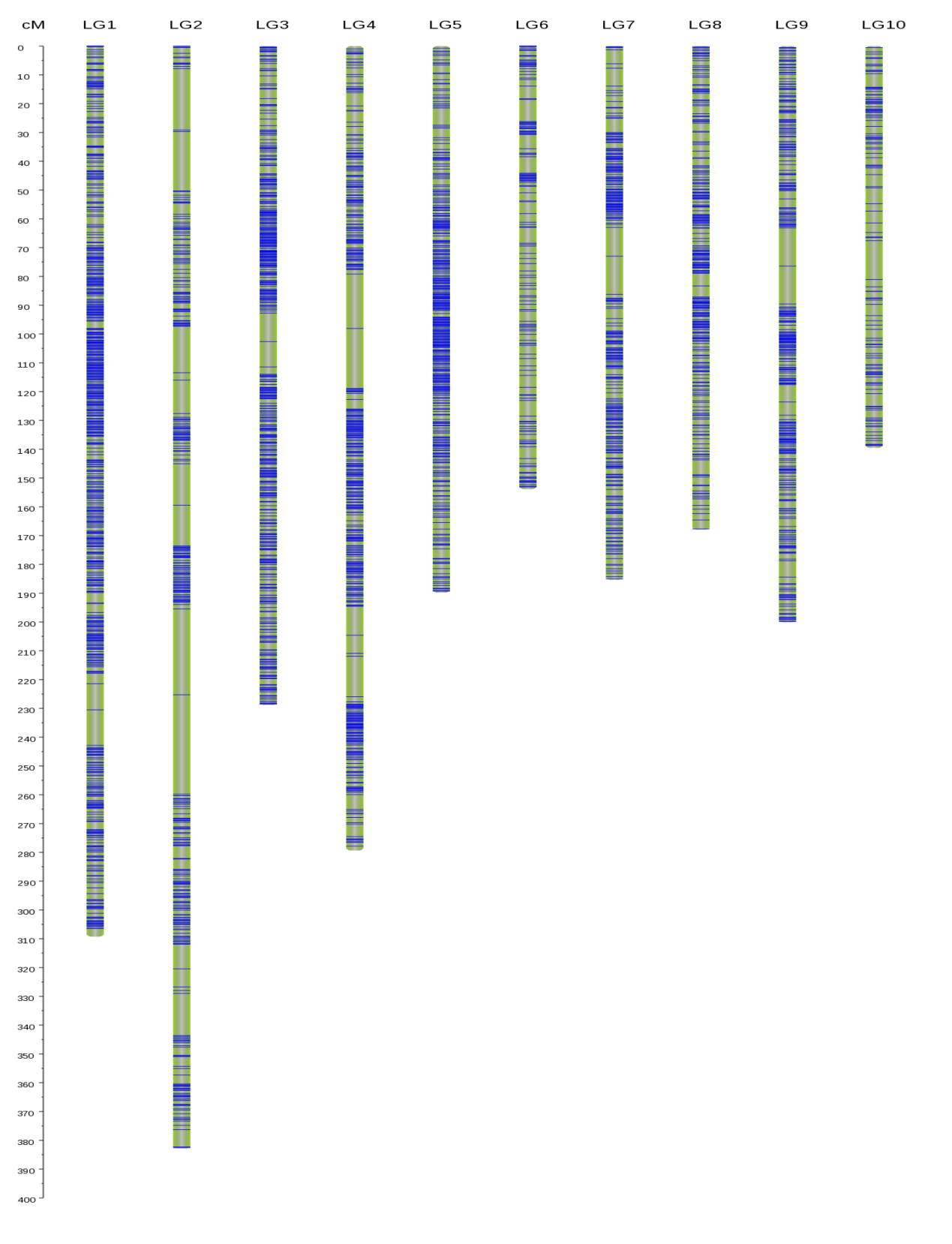


**Figure S4**. Intra-specific genetic linkage map of maize constructed using the F_2_ population derived from the cross of SG7 and SG5.
