# Supplementary material for: High Density Linkage Map Construction and Mapping of Yield Trait QTLs in Maize (Zea mays) Using the Genotyping-by-Sequencing (GBS) Technology
Source: Front Plant Sci. 2017 May 8;8:706. doi: 10.3389/fpls.2017.00706 (PMC5420586; doi:10.3389/fpls.2017.00706)
Supplement: Supplementary file 5 [file DataSheet5.DOCX]

P2

P1

P1

P2

P2

P1

P1

P1

P2

P1

P2

P2

P2

P1

P2

P1

P2

P1


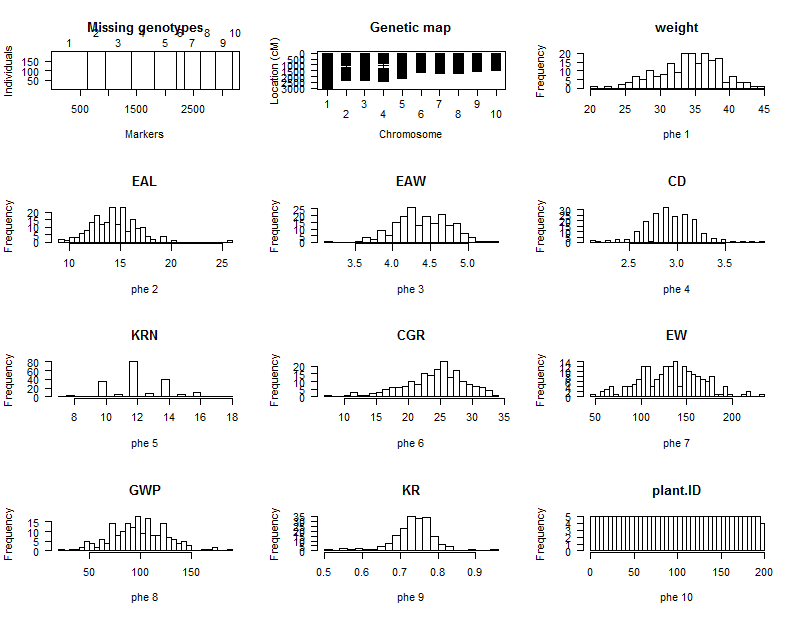

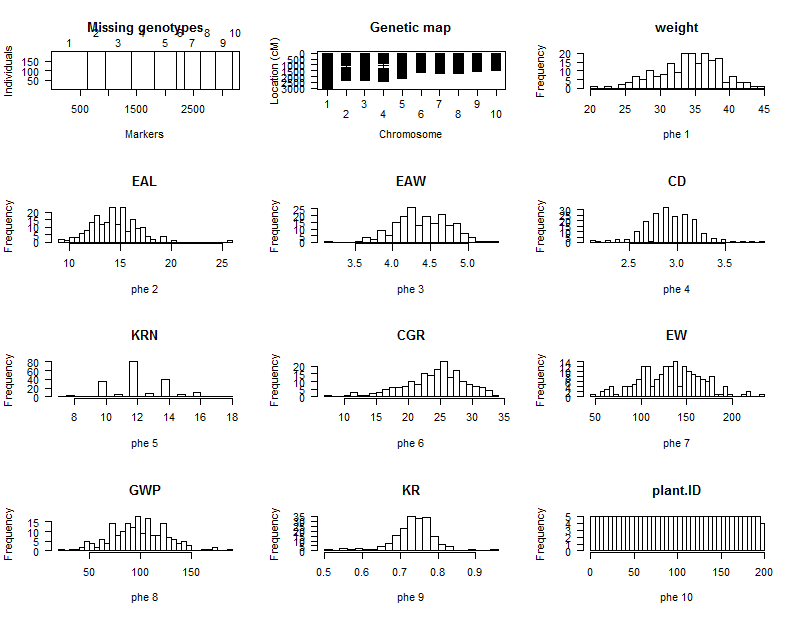

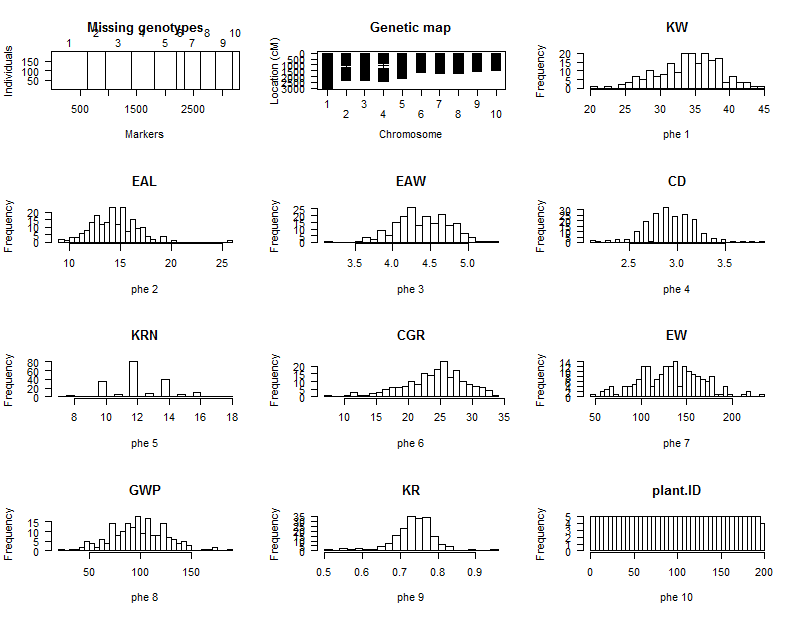

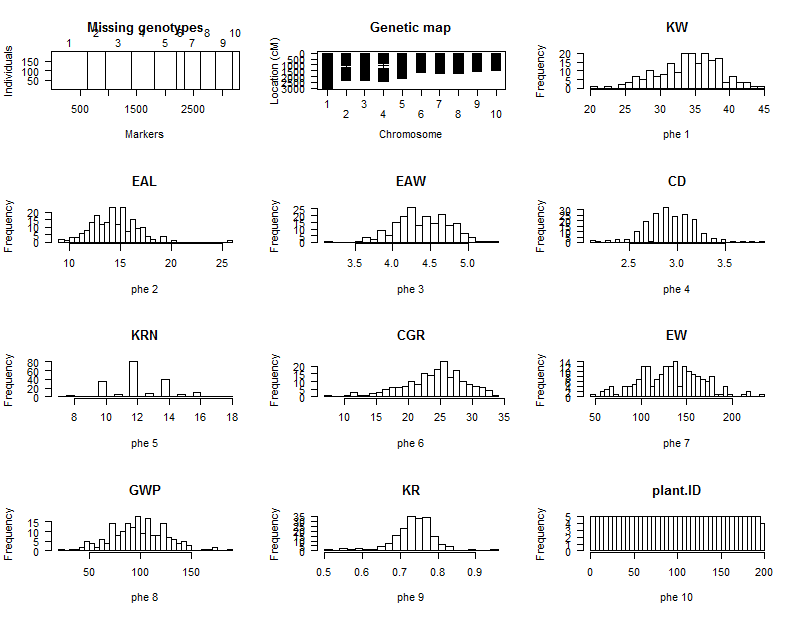


**Figure S5.** Distribution of the ear length (EAL), ear width (EAW), cob diameter (CD), kernel row number (KRN), corn grains per row (CGR), ear weight (EW), grain weight per plant (GWP), kernel ratio (KR) and 100-kernel weight (KW) in the F_2_ population derived from the cross of SG5 and SG7. P1 and P2 indicate the two parents, SG5 and SG7, respectively.
