# Supplementary material for: High Density Linkage Map Construction and Mapping of Yield Trait QTLs in Maize (Zea mays) Using the Genotyping-by-Sequencing (GBS) Technology
Source: Front Plant Sci. 2017 May 8;8:706. doi: 10.3389/fpls.2017.00706 (PMC5420586; doi:10.3389/fpls.2017.00706)
Supplement: Supplementary file 6 [file Table1.DOCX]

**Table S1**. Eight types of SNP markers between two parental lines of maize

| Marker type | Numbers | Percentage (%) |
| --- | --- | --- |
| ccxab | 73 | 0.05 |
| nnxnp | 27,967 | 20.88 |
| lmxll | 23,616 | 17.63 |
| aaxbb | 68,882 | 51.43 |
| abxcd | 0 | 0.00 |
| hkxhk | 13,054 | 9.75 |
| abxcc | 75 | 0.06 |
| efxeg | 269 | 0.20 |
| Total number | 133,936 | 100.00 |
