# Supplementary material for: High Density Linkage Map Construction and Mapping of Yield Trait QTLs in Maize (Zea mays) Using the Genotyping-by-Sequencing (GBS) Technology
Source: Front Plant Sci. 2017 May 8;8:706. doi: 10.3389/fpls.2017.00706 (PMC5420586; doi:10.3389/fpls.2017.00706)
Supplement: Supplementary file 7 [file Table2.DOCX]

**Table S2**. Numbers and frequencies of SNPs detected on individual chromosomes of maize

| Chromosome | < 5cM | 5 cM to 10 cM | 10 cM to 20 cM | > 20 cM | Percentage (%) |
| --- | --- | --- | --- | --- | --- |
| 1 | 621 | 1 | 1 | 0 | 99.68 |
| 2 | 311 | 3 | 5 | 4 | 96.29 |
| 3 | 447 | 2 | 0 | 0 | 99.56 |
| 4 | 408 | 2 | 3 | 1 | 98.55 |
| 5 | 379 | 1 | 0 | 0 | 99.74 |
| 6 | 136 | 4 | 0 | 0 | 97.14 |
| 7 | 287 | 2 | 1 | 0 | 98.97 |
| 8 | 251 | 1 | 0 | 0 | 99.60 |
| 9 | 302 | 2 | 2 | 0 | 98.69 |
| 10 | 117 | 1 | 1 | 0 | 98.32 |
| Total | 3,259 | 19 | 13 | 5 | 98.65 |
