# Supplementary material for: High Density Linkage Map Construction and Mapping of Yield Trait QTLs in Maize (Zea mays) Using the Genotyping-by-Sequencing (GBS) Technology
Source: Front Plant Sci. 2017 May 8;8:706. doi: 10.3389/fpls.2017.00706 (PMC5420586; doi:10.3389/fpls.2017.00706)
Supplement: Supplementary file 9 [file Table4.DOCX]

**Table S4.** Summary statistics and distributions of gaps of the maize intra-specific genetic linkage map constructed using F_2_ individuals arising from the cross of SG7 and SG5.

| Chromosome | Length | Genes | Exons | Raw SNPs | Raw SNPs/100 kb | Filtered SNPs |
| --- | --- | --- | --- | --- | --- | --- |
| A01 | 301476924 | 6003 | 27545 | 457898 | 151.88 | 4643 |
| A02 | 237917468 | 4741 | 21358 | 353110 | 148.42 | 3619 |
| A03 | 232245527 | 4171 | 19359 | 352237 | 151.67 | 4017 |
| A04 | 242062272 | 4179 | 18546 | 364833 | 150.72 | 3999 |
| A05 | 217959525 | 4473 | 20930 | 298791 | 137.09 | 3459 |
| A06 | 169407836 | 3276 | 15022 | 228761 | 135.04 | 515 |
| A07 | 176826311 | 3114 | 14182 | 252254 | 142.66 | 3012 |
| A08 | 175377492 | 3505 | 16059 | 266248 | 151.81 | 3140 |
| A09 | 157038028 | 2989 | 13586 | 241358 | 153.69 | 3237 |
| A10 | 149632204 | 2688 | 12041 | 231598 | 154.78 | 240 |
